# Supplementary material for: Comparative Effect of Two Types of Physical Exercise for the Improvement of Exercise Capacity, Diastolic Function, Endothelial Function and Arterial Stiffness in Participants with Heart Failure with Preserved Ejection Fraction (ExIC-FEp Study): Protocol for a Randomized Controlled Trial
Source: J Clin Med. 2023 May 18;12(10):3535. doi: 10.3390/jcm12103535 (PMC10218785; doi:10.3390/jcm12103535)
Supplement: Supplementary file 1 [file jcm-12-03535-s001.zip › jcm-2282641-supplementary.pdf]

Supplementary file

Table S1. Basic clinical features.

Primary outcomes					Covariates							
**Exercise capacity**	**Echocardiography**	**Endothelial function**	**Arterial stiffness**	**Quality of life**	**Sociodemographic variables**	**Patient medical history**	**Anthropometric variables**	**Muscle strength**	**Spirometry**	**Biochemical parameters**	**Physical activity**	**Mediterranean diet adherence**
VO2max (ml/min/kg)	E velocity (m/s)	cIMT (mm)	PWv (m/s)	SF-12	Age (years)	Comorbidities (n, %)	Weight (kg)	Handgrip strength (kg)	FVC (L)	Glucose (mg/dL)	Accelerometry (g)	MEDAS-14
Workload (watts)	A velocity (m/s)		cAIx (%)	MLWHFQ	Sex	Pharmacological treatment	Height (cm)		FEVI (L)	Total cholesterol (mg/dL)	Podometry (steps/day)	
Exercise time (s)	E/A ratio				Socioeconomic level		BMI (kg/m2)		FEVI/FVC (%)	Triglycerides (mg/dL)		
HRmax (beats/min)	e' velocity ratio						Densitometry			HDL cholesterol (mg/dL)		
6-minute walking test (m)	Ejection fraction percentage (%)						Blood pressure (mmHg)			LDL cholesterol (mg/dL)		
	Left ventricular volume index (mL/m2)									Apolipoproteins A1 and B (mg/dL)		
	End-diastolic volume (mL)									Insulin (mlU/L)		
	Left ventricular mass (g)									Ultrasensitive C-reactive protein (mg/L)		
	Left atrial diameter (mm)									NT-proBNP (pg/mL)		
	Isovolume relaxation time (ms)									HbA1c (%)		
	Deceleration time (ms)											
	Left atrial volume index											
